# Anthocyanin and Phenolic Acids Contents Influence the Color Stability and Antioxidant Capacity of Wine Treated With Mannoprotein

**DOI:** 10.3389/fnut.2021.691784

**Published:** 2021-06-18

**Authors:** Xiao-feng Yue, Si-si Jing, Xiao-fan Ni, Ke-kun Zhang, Yu-lin Fang, Zhen-wen Zhang, Yan-lun Ju

**Affiliations:** ^1^College of Enology, Northwest A&F University, Xianyang, China; ^2^Shaanxi Engineering Research Center for Viti-Viniculture, Xianyang, China; ^3^Heyang Viti-Viniculture Station, Northwest A&F University, Xianyang, China

**Keywords:** antioxidant capacity, color stability, mannoprotein, wine, phenolic acids

## Abstract

Wine is consumed by humans worldwide, but the functional components are lost and the color changes during its production. Here, we studied the effects of mannoprotein (MP) addition (0, 0.1, and 0.3 g/L) upon crushing and storage. We measured anthocyanins, phenolic acids profiles, color characteristics, and antioxidant activities of wine. The results showed that the addition of MP before fermentation significantly increased the total phenolic content (TPC), total anthocyanin content, total tannin content (TTC), total flavonoid content, and total flavanol content in wine, whereas the addition of MP during storage had the opposite effect. The addition of MP before alcohol fermentation significantly increased the amount of individual anthocyanins and individual phenolic acids, maintained the color, and increased the antioxidant capacity of wine. In addition, the addition of 0.3 g/L MP during storage increased the content of individual phenolic acids and TPC of wine. However, the addition of 0.1 g/L MP during storage significantly reduced the TPC, TAC, TTC, and individual anthocyanin content (except for malvidin-3-glucoside and malvidin-3-acetly-glucoside); meanwhile, the treatment attenuated the color stability and antioxidant capacity of wine. The results demonstrated that the addition of MP before alcohol fermentation could increase the functional components and improve the color stability and antioxidant capacity of wine.

## Introduction

Wine directly affects the human nervous system and increases muscle tension, improves human immunity, and scavenges reactive oxygen species (ROS) to protect cells from oxidative damage. The most important bioactive components in wine are anthocyanins, phenolic acids, flavonoids, tannins, and vitamins (1).

Anthocyanins are natural pigment formed by the combination of anthocyanidin and sugar via a glycosidic bond. They are non-toxic and widely present in the cellular fluid of plant flowers, fruits, stems, leaves, and other organs leading to coloration. They also have many health functions for the human body such as antioxidant activity, anti-tumor, anti-cancer, and anti-inflammatory. They inhibit lipid peroxidation and have been used in food, health products, cosmetics, medicine, and other industries (2–4). For grape berries and wines, anthocyanins are one of the most important bioactive components, and anthocyanins can provide color and antioxidant activity for wine; the composition and content of anthocyanins of wine play important roles in its color stability (5).

Phenolic acids are another important bioactive component in wine. Phenolic acids also have strong antioxidant function and play an important role in the stability and taste of wine. The anthocyanins and phenolic acids in wine are mainly from grape berries; however, their stability and composition are affected by many factors, including pH, yeast, storage temperature and time, and light exposure during the fermentation process (6, 7). Very strong light will cause degradation of anthocyanins and phenolic acids in wine. Changes in the composition and contents of anthocyanins and phenolic acids not only affect the color stability and sensory quality but also affect the nutritional value of wine and ultimately affect its consumption (8, 9). Thus, research on effective preservation techniques for anthocyanins and phenolic acids in wine is urgently required in viticulture.

Mannoprotein (MP) is usually used in winemaking to protect the stability of wine color. The interaction between MP and phenolic acids or anthocyanins in wine can affect the sensory quality and color stability of wine and has attracted the interest of researchers (10–12). However, the main drawback of existing research is that studies have focused only on the effect of MP on wine quality during wine storage post-fermentation (13–16), and ignores the influence of adding MP to the grape must before fermentation, as the anthocyanins and phenolics in wine come from the fermentation and maceration of grape must. In view of this, we speculate that the addition of MP to grape also has a good protective effect on the color stability and antioxidant capacity of wine. The results of this study will provide a theoretical basis for the use of MP before alcohol fermentation and enrich the scope of MP use in winemaking.

Thus, the objective of this study was to evaluate the effects of different concentrations of MP addition at crushing and storing on anthocyanins, phenolic acids profiles, color stability, and antioxidant activities of Cabernet Sauvignon red wine. According to previous reports, the most commonly used concentrations of MP in wine research are near 0.1, 0.2, 0.25, and 0.3 g/L, with most positive effects seen from treatments with concentrations between 0.1 and 0.3 g/L (17, 18). Therefore, concentrations of 0.1 and 0.3 g/L MP were used in this study.

## Materials and Methods

### Grapes and Wine Samples

Cabernet Sauvignon (*V. Vinifera* L.) grapes were collected from Lilan winery a commercial vineyard in Yinchuan, NingXia, China, and used as materials. Grapes were harvested for winemaking when the total sugar content reached 230–240 g/L. For MP (MP30, Yeast extract, Biolees, Laffort, Bordeaux, France) treatment at crushing, grapes were crushed and destemmed, and the must was mixed with sulfur dioxide (SO_2_, 50 mg/L), pectinase (30 mg/L, Laffort, Bordeaux, France), yeast (200 mg/L, Rhone 2323, Lalvin, Denmark), and MP (0, 0.1, and 0.3 g/L, respectively). The alcohol fermentation was carried out in 100-L stainless-steel tanks and held at 25°C ± 1°C for 12 days. For MP treatment at storing, wine samples fermented without MP treatment were collected and mixed with MP (0, 0.1, or 0.3 g/L). All wine samples were stored at 20°C for 12 months until analysis.

### Reagents and Standards

Methanol, ethyl acetate, acetic acid, and acetonitrile (all HPLC-grade) were purchased from Fisher (Suwanee, GA, USA). Folin-Ciocalteu, 6-hydroxy-2,5,7,8-tetramethylchroman-2-carboxylic acid (Trolox) and 2,2-diphenyl-1-picrylhydrazyl (DPPH) (analytical grade) were obtained from Sigma (Shanghai, China). The standards were purchased from Sigma (Shanghai, China), namely, malvidin-3-5-*O*-diglucoside (≥95%, HPLC), gallic acid (≥98%, HPLC), proanthocyanidin B1 (≥95%, HPLC), protocatechin (≥99%, HPLC), chlorogenic acid (≥95%, HPLC), catechin (≥98%, HPLC), proanthocyanidin B2 (≥98%, HPLC), epicatechin (≥98%, HPLC), caffeic acid (≥95%, HPLC), coumaric acid (≥98%, HPLC), rutin (≥98%, HPLC), ferulic acid (≥99%, HPLC), quercetin-3-D-β-glucoside (≥98%, HPLC) myricetin (≥98%, HPLC), quercetin (≥98%, HPLC), and kaempferol (≥95%, HPLC).

### Determination of Physicochemical Indices of Wine

The determination of the reducing sugar, total acidity, and alcohol content of wine samples was conducted according to the methods of OIV (19). The measurement of total phenolic content (TPC) was performed by the Folin-Ciocalteu colorimetric method reported previously (20). The pH differential method was used to measure the total anthocyanin content (TAC) (21). The total tannin content (TTC) was estimated by the methylcellulose precipitation (MCP) method (22). The total flavonoid content (TFC) and total flavanol content (TFAC) were detected according to Meng et al. (20).

### HPLC-MS/MS Analysis of Anthocyanin Profiles

The determination of anthocyanin profiles in wine samples was according to our previous reports (23, 24). Briefly, the anthocyanin profiles were analyzed by a high performance liquid chromatography (HPLC) system (Shimadzu Co., Ltd., Kyoto, Japan) fitted with a C_18_ column (250 × 4.6 mm; Shimadzu Co., Ltd.). Phase A was formic acid: acetonitrile: water (7:10:83, *v/v/v*); phase B was formic acid: acetonitrile: water (2:54:44, *v/v/v*). Wine samples were filtered with a 0.22 μm filter before injection. The sample injection amount was 20 μL. The wine samples were eluted using phase B, flowing by 0–30% for 15 min with 1.0 mL/min flow rate; 30–50% phase B for 10 min; and then 50% phase B for 10 min. The anthocyanins were detected at 525 nm and scanned at 200–600 nm.

The mass spectrometric (MS) acquisition parameters were as follows: electrospray ionization (ESI) interface and negative ion model; nitrogen was used as drying and nebulizing gas and nebulizer pressure was 380 Pa; 10 mL/min dry gas flow rate, 325°C dry gas temperature, and scanned at 100–1,000 m/z. The identification of anthocyanins were according to their order of elution and retention time (RT) with respect to malvidin-3,5-*O*-diglucoside and the weight of the molecular ion and the fragment ion compared with standards and references (25, 26).

Anthocyanins were quantified using malvidin-3,5-*O*-diglucoside (Sigma, Shanghai, China, purity ≥95%; *y* = 0.00002*x* + 0.3689, *R*^2^ = 0.9999) as the standard according to the reported methods (20, 24, 27). The concentrations of individual anthocyanins were expressed as malvidin-3,5-*O*-diglucoside equivalence (ME, mg/L) based on the standard calibration curve. The equation was: Concentration (mg/L) = 0.003 * Area. The calculation process taken into account the influence of Molecular weights (MW) on the calculation results, so the concentration obtained was multiplied by the MW of the individual anthocyanin and dividing by the MW of malvidin-3,5-*O*-diglucoside chloride (MW = 691.5). The final equation was: Concentration (mg/L) = 0.003 * Area * MW/691.5 (27).

### Analysis of Phenolic Acids Profiles

#### Extraction of Phenolic Acids

The extraction of phenolic acids in wine samples was according to previous reports with some modify (28). Briefly, the samples were mixed with water and ethyl acetate (V/V/V, 1:1:0.8), shaken, and allowed to stand for 30 min. We then collected the upper organic phase and extracted them three times. The organic phase was evaporated to dryness with a rotary evaporator under 33°C, and the residue was then re-dissolved with 5 mL methanol (HPLC grade). The extractions were filtered with a 0.45 μm filter before HPLC analysis.

#### Determination of Phenolic Acids Profiles

The identification and quantification of individual phenolic acids from wine samples were performed by HPLC (Shimadzu Co., Ltd.) equipped with a C_18_ column (250 × 4.6 mm; Shimadzu Co., Ltd.) and a VWD detector. The initial temperature of the column was 30°C. The injection amount of sample extraction was 10 μL. Mobile phase A comprised 1% acetic acid aqueous solution and phase B comprised 1% acetic acid acetonitrile aqueous solution. The samples were eluted using phase B, 5–25% for 40 min with 1.0 mL/min flow rate; 25–35% phase B for 5 min; and then 35–50% phase B for 5 min. The detection wavelength was 280 nm. The identification and quantification of phenolic acids were according to the retention times and the calibration curves of their standards.

### Wine Color Measurement

A CR400 chrominometer (Konica Minolta, Inc., Japan) was used to determine the values of L^*^, a^*^, and b^*^ with a chrominometer whiteboard color as the standard (29). The value of C was expressed as C = (a^*2^ + b^*2^)^1/2^, H = arctan (b^*^/a^*^). The value of ΔE was expressed as ΔE = [(ΔL)^2^ + (Δa^*^)^2^ + (Δb^*^)^2^]^1/2^.

### Co-pigmentation Effect

The magnitude of the co-pigmentation (M) was determined by a UV-2450 spectrophotometer (Shimadzu, Japan) at 520 nm according to the methods of Sun et al. (18). The value of M was expressed as M = [(A–A_0_)/A_0_] × 100. A and A_0_ represent the absorbance value of wine samples in the MP treatment group and the control group, respectively.

### Analysis of Antioxidant Capacity

Three different methods were used to estimate antioxidant capacity: 1,1-diphenyl-2-picrylhydrazyl (DPPH), ferric reducing antioxidant power (FRAP), and hydroxyl radical scavenging ability (HRCA). For the determination of DPPH, a UV-2450 spectrophotometer (Shimadzu, Japan) was used to measure the absorbance against a negative control at 517 nm. The DPPH value was expressed as DPPH (%) = [(A_blank_ – A_sample_)/A_blank_] × 100%. To measure the HRCA, different concentrations of wine samples were mixed with FeSO_4_ (6 mmol/L, 0.5 mL), salicylic acid (6 mmol/L, 0.5 mL), H_2_O_2_ (0.5 mL), and ddH_2_O to obtain a 6 mL reaction system. They then reacted in a water bath at 37°C for 1 h, and the absorbance was measured at 510 nm. A HRCA value was expressed as HRCA (%) = [1–(A_sample_ – A_control_)/A_blank_] × 100%. For the measurement of FRAP, a 1 mL wine sample was reacted with 5 mL TPTZ (tripyridyltriazine) in a 37°C water bath for 10 min. We then measured the absorbance at 593 nm. The results were expressed as FeSO_4_ equivalence (μmol/L), using the FeSO_4_ × 7H_2_O calibration curve at the concentration ranged from 100 to 1,000 μmol/L.

### Statistical Analysis

All results were expressed as mean ± SD (*n* = 3). SPSS 21.0 software (SPSS Inc., Chicago, IL, USA) was used to perform the one-way ANOVA test by Duncan's test, and *p* < 0.05 was set as significant level. MetaboAnalyst 5.0 (http://www.metaboanalyst.ca/) was employed to carry out the multivariate statistical analysis.

## Results and Discussion

### Physicochemical Characteristics

The effects of MP addition on the physicochemical characteristics of wine including reducing sugar, total acidity, alcohol, TPC, TAC, TTC, TFC, and TFAC were measured in each sample. Supplementary Figure 1 shows that the addition of MP to the grape must occur before alcohol fermentation has a significant effect on the reducing sugar, titratable acid, and alcohol content of the wine. Compared with the control group, the addition of 0.1 g/L MP significantly reduced the titratable acid, reducing sugar, and alcohol content of wine. Previous studies showed that MP could reduce the contents of malic acid and lactic acid in wine during malolactic fermentation, thereby reducing the content of wine titratable acid (30). In addition, studies reported that MP could slightly increase the sugar content of alcoholic products, which in turn had an effect on alcohol content possibly because MP is a polysaccharide (20). The present study found that different concentrations of MP had different effects on reducing sugar and alcohol content of wine. The addition of 0.3 g/L MP before alcohol fermentation significantly increased the reducing sugar and alcohol content, whereas 0.1 g/L MP had the opposite effect. However, the effect of MP addition before alcohol fermentation on the physicochemical characteristics of wine—in particular how different concentrations of MP affect wine quality—was not clearly indicated; thus, further research is needed.

Table 1 shows the effects of mannoprotein concentration on the polyphenols of wine, that is, TPC, TAC, TTC, TFC, and TFAC, at different stages of fermentation. Compared with the control group, the addition of different concentrations of MP before alcohol fermentation significantly increased the phenolic content of the wine. The effect on TTC was the most significant—it was 2.35 and 3.43 times that of the control group—followed by TPC, which was 1.21 and 1.15 times that of the control group. The addition of MP during the storage process significantly reduced the phenolic content in wine except for an insignificant effect on the TFAC. Previous studies reported that MP could affect the phenolic content of wine and blueberry wine during storage, thereby affecting its color stability and antioxidant activity (13, 20). This study indicated that adding MP before alcohol fermentation was better than adding it after fermentation and could significantly increase the phenolic content in wine.

**Table 1:** Effects of mannoprotein concentration on the polyphenols of wine at different stages of fermentation (mg/L).

	**CK**	**Mannoprotein treatment (g/L)**			
		**Before fermentation**		**After fermentation**	
		**0.1**	**0.3**	**0.1**	**0.3**
TPC	190.52 ± 0.43d	229.872 ± 0.32a	218.232 ± 0.82b	80.274 ± 0.04e	205.933 ± 0.79c
TAC	348.491 ± 0.82b	376.299 ± 0.49a	370.362 ± 2.25a	204.852 ± 0.38c	204.557 ± 0.70c
TTC	232.571 ± 0.85c	546.671 ± 5.19b	797.602 ± 1.06a	97.541 ± 2.41e	182.406 ± 0.84d
TFC	86.930 ± 0.71b	96.421 ± 0.86a	85.201 ± 0.06b	85.380 ± 0.92b	75.300 ± 0.63c
TFAC	0.570 ± 0.12c	0.790 ± 0.14b	1.090 ± 0.15a	0.580 ± 0.02c	0.580 ± 0.00c

*Different letters in the row indicate significant differences (Duncan test, p < 0.05) among treatments*.

*CK, control group with 0 g/L MP; TPC, total polyphenols content; TAC, total anthocyanins content; TTC, total tannin content; TFC, total flavonoids content; TFAC, total flavanols content*.

### Anthocyanin Profiles of Wine Samples

Anthocyanins play an important role in wine color stability and sensory quality and are important functional components in wine (9). Here, the concentration and composition of anthocyanins in wine were tested to evaluate the effect of MP addition on wine color stability and antioxidant capacity. For a better characterization of the anthocyanins profiles of wines used in the present study, a detailed study by HPLC-MS/MS was performed. A total of nine anthocyanins were investigated and analyzed: Delphinidin-3,5-*O*-diglucoside, Cyanidin-3,5-*O*-diglucoside, Petunidin-3,5-*O*-diglucoside, Peonidin-3,5-*O*-diglucoside, Malvidin-3,5-*O*-diglucoside, Petunidin-3-*O*-acetly-5-*O*-glucoside, Malvidin-3-*O*-acetly-5-*O*-glucoside, Peonidin-3-*O*-coumayl-5-*O*-glucoside, Malvidin-3-*O*-coumayl-5-*O*-glucoside (Table 2 and Supplementary Figure 2). The retention times, molecular ion masses, basic MS^2^ fragments of each anthocyanin were shown in Table 2. Although some of the above anthocyanins were co-eluted under the applied chromatographic conditions, it was possible to identify all compounds by extracting ion chromatograms obtained in MS^2^ mode for each group of anthocyanins with the same aglycone (anthocyanin) at the m/z characteristic values of each anthocyanidin (Table 2). Compared with the control group, the addition of 0.1 g/L and 0.3 g/L MP before alcohol fermentation significantly increased the contents of petunidin-3-5-*O-*diglucoside and malvidin-3-5-*O-*diglucoside in wine but had no significant effect on other anthocyanins; the addition of 0.1 g/L MP during storage significantly improved the contents of delphinidin-3-5-*O*-diglucoside, cyanidin-3-5-*O*-diglucoside, petunidin-3-5-*O*-diglucoside, peonidin-3-5-*O*-diglucoside, peonidin-3-*O*-coumayl-5-*O*-glucoside, and malvidin-3-*O*-coumayl-5-*O*-glucoside. It significantly reduced the contents of malvidin-3-5-*O*-diglucoside, petunidin-3-*O*-acetly-5-*O*-glucoside, and malvidin-3-*O*-acetly-5-*O*-glucoside. The addition of 0.3 g/L MP during storage significantly increased the contents of cyanidin-3-5-*O-*diglucoside and petunidin-3-5-*O-*diglucoside and significantly reduced the contents of peonidin-3-5-*O-*diglucoside, malvidin-3-5-*O-*diglucoside, petunidin-3-*O*-acetly-5-*O*-glucoside, and malvidin-3-*O*-acetly-5-*O*-glucoside. Interestingly, the addition of MP during storage significantly increased the contents of cyanidin-3-5-*O-*diglucoside and petunidin-3-5-*O-*diglucoside reaching the highest levels of 16.22 and 79.62 mg/L, respectively; however, it significantly reduced the contents of malvidin-3-5-*O-*diglucoside, which was only 0.24–0.25 mg/L. The results indicated that the addition of MP before fermentation was more helpful than the addition of MP at storage to increase the anthocyanin contents in wine.

**Table 2:** Effects of mannoprotein concentration on the anthocyanins profiles of wine at different stages of fermentation (mg/L).

**Retention time *t*_**R**_ (min)**	**Anthocyanins**	**[M]^**+**^ (Fragment MS^**2**^, m/z)**	**Ref(s)**	**CK**	**Mannoprotein treatment (g/L)**				
					**Before fermentation**			**After fermentation**	
					**0.1**	**0.3**		**0.1**	**0.3**
5.54	Delphinidin-3,5-*O*-diglucoside	465 (303)	(26, 31, 32)	9.950 ± 0.14bc	11.220 ± 0.09b	11.140 ± 0.07b		14.540 ± 0.07a	7.760 ± 0.40c
8.74	Cyanidin-3,5-*O*-diglucoside	449 (287)	(26, 31, 32)	1.490 ± 0.03c	2.410 ± 0.06c	1.620 ± 0.00c		16.220 ± 0.10a	6.310 ± 0.33b
10.15	Petunidin-3,5-*O*-diglucoside	641 (479, 317)	(26, 31, 32)	14.810 ± 0.17e	16.760 ± 0.03c	15.130 ± 0.05d		79.620 ± 0.41a	79.280 ± 0.07b
14.01	Peonidin-3,5-*O*-diglucoside	625 (301)	(26, 31, 32)	4.350 ± 0.39b	4.270 ± 0.01b	4.650 ± 0.21b		7.800 ± 0.04a	3.260 ± 0.16c
15.52	Malvidin-3,5-*O*-diglucoside	655 (331, 493)	(26, 31, 32)	193.862 ± 0.24c	195.480 ± 0.36b	201.780 ± 0.10a		0.240 ± 0.00d	0.250 ± 0.04d
30.39	Petunidin-3-*O*-acetly-5-*O*-glucoside	683 (479, 317)	(26)	4.360 ± 0.10b	4.510 ± 0.04a	4.440 ± 0.01ab		0.260 ± 0.00d	0.530 ± 0.19c
31.70	Malvidin-3-*O*-acetly-5-*O*-glucoside	697 (535, 493, 331)	(26)	66.740 ± 0.96a	67.150 ± 0.32a	65.880 ± 0.17b		1.110 ± 0.00c	1.180 ± 0.52c
41.69	Peonidin-3-*O*-coumayl-5-*O*-glucoside	771 (625, 463)	(26, 31, 32)	1.600 ± 0.22b	2.030 ± 0.04b	1.590 ± 0.02b		3.900 ± 0.01a	2.090 ± 0.11b
42.32	Malvidin-3-*O*-coumayl-5-*O*-glucoside	801 (655, 493, 331)	(26, 31, 32)	14.351 ± 0.10b	15.420 ± 0.36b	11.280 ± 0.17c		17.440 ± 0.03a	14.622 ± 0.92b
	Total			311.51	319.25	317.51		182.71	115.28

*Different letters in the row indicate significant differences (Duncan test, p < 0.05) among treatments*.

*CK, control group with 0 g/L MP*.

### Wine Color Characteristics

**Figure 1:**
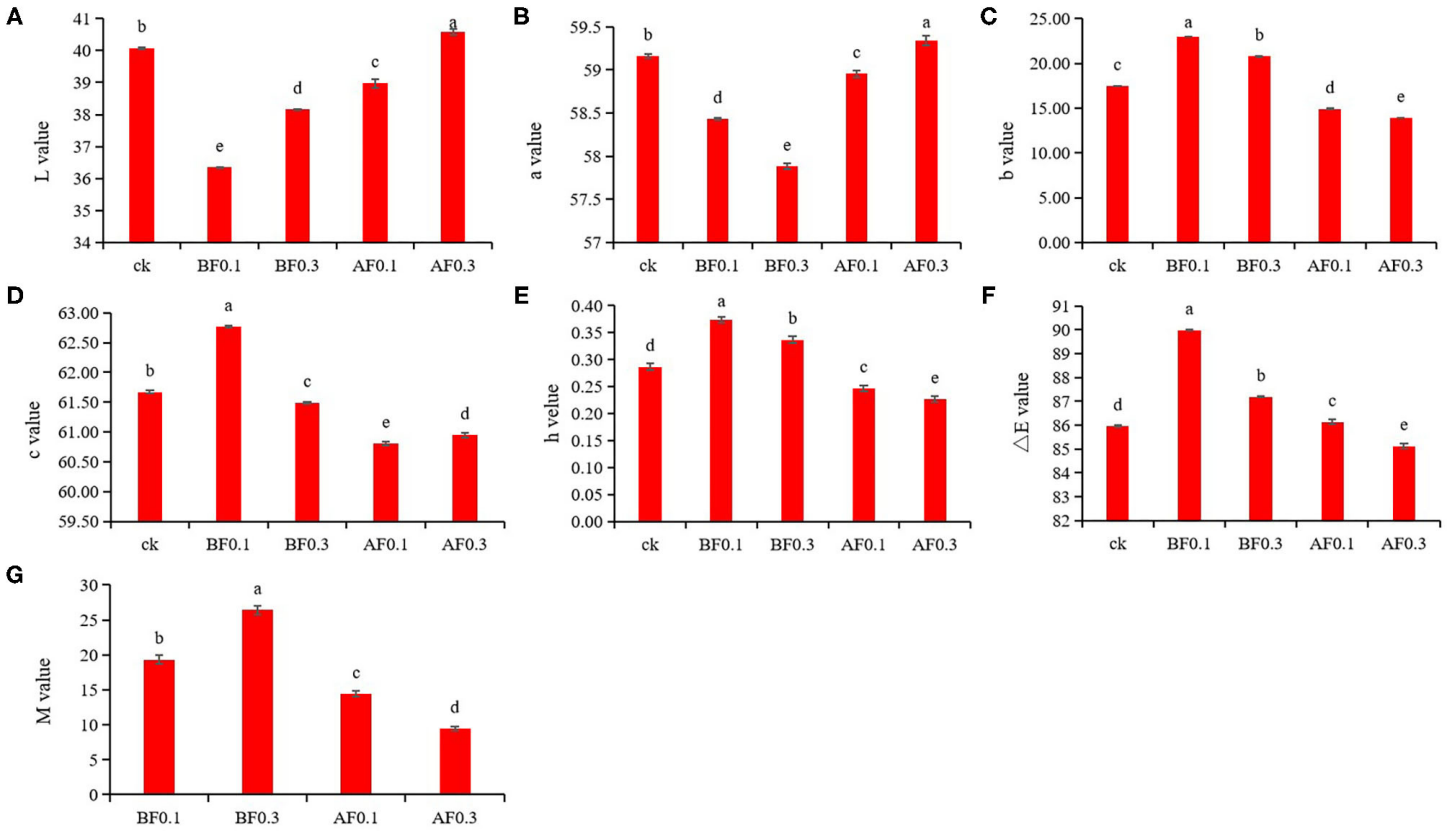
Effect of mannoprotein concentration on the color characteristics of wine. **(A)**: L* value, **(B)**: a* value, **(C)**: b* value, **(D)**: c* value, **(E)**: h* value, **(F)**: ΔE value, and **(G)**: copigmentation value M. Values presented are means ± SD (*n* = 3). Different letters indicate significant differences among treatments using Duncan test (*p* < 0.05). CK: control group with 0 g/L MP, BF0.1: 0.1 g/L mannoprotein addition before fermentation, BF0.3: 0.3 g/L mannoprotein addition before fermentation, AF0.1: 0.1 g/L mannoprotein addition after fermentation, BF0.3: 0.3 g/L mannoprotein addition after fermentation.

To analyze the influence of the addition of MP on wine color stability, CIELAB color space was performed to analyze the wine color characteristics. Figure 1 shows that the MP treatment significantly changed the value of ΔE vs. control indicating that the MP treatment had a significant effect on the wine color (29). In this study, wine samples treated with 0.1 g/L or 0.3 g/L MP before alcohol fermentation and 0.1 g/L MP during storage showed lower L^*^ and a^*^ values indicating that these wines had a darker color and lighter red intensity. The wine samples treated with 0.3 g/L MP during storage exhibited the lightest color and deepest red intensity with the highest L^*^ and a^*^ values. The wine samples treated with 0.1 g/L MP before alcohol fermentation exhibited the strongest yellow color and a brighter chroma, as these wines had the highest positive b^*^, C, and H values. However, a lighter yellow color was observed in wine samples treated with 0.1 and 0.3 g/L MP during storage; they had a lower positive b^*^ and H values. Previous studies also found that the addition of MP after alcohol fermentation significantly decreased wine color density (33).

For co-pigmentation (M) value, the addition of 0.1 and 0.3 g/L MP before alcohol fermentation significantly increased the value of M, which suggested that the addition of MP in grape must leads to a deeper color in wine samples (20, 34). Previous researchers found that adding MP during storage could increase the M value of blueberry wine, which was slightly different from the results of this study. It might due to the addition of MP could have different effects on the contents of anthocyanins, phenols and tannins of blueberry wine and wine, respectively (20, 35). These results showed that the addition of MP before fermentation could give wine a deep and bright red color, which is attractive to consumers and was helpful for wine color stability.

### Phenolic Acids Profiles of Wine Samples

Polyphenols have an important contribution to the astringency, bitterness, structure, and antioxidant capacity of wine. Polyphenols play an important role in the stability of wine color during wine storage (36). The polyphenols in wine mainly come from grape skins and seeds through maceration. However, the phenolic components are easily affected by the temperature, pH, yeast strains, and other factors during the alcohol fermentation and storage (6, 7). MP can protect the polyphenols from degradation. Previous studies have found that MP could affect the interactions of polyphenols (such as flavanols) with proteins by which could modulate the astringency and color stability of wine (13, 16, 37), and those interactions were not only affected by the composition of MP but also the structure of flavanols (37). Therefore, it is precisely because of this interaction that the polyphenols were protected from degradation.

This study analyzed the effects of MP addition in the grape must as well as the storage process on the phenolic components in wine (Table 3) and the chromatogram of the phenolic acids standards was shown in Supplementary Figure 3. Compared with the control group, the addition of MP before alcohol fermentation significantly increased the total amount of phenolic acids in the wine, particularly in the 0.1 g/L MP treatment group, in which the phenolic content in wine reached 1.22 times that of the control group. Here, the contents of proanthocyanidin B2 and proanthocyanidin B1 were the highest, reaching 200.39 and 158.22 mg/L in the 0.1 g/L MP treatment group, respectively. The addition of 0.1 g/L MP before alcohol fermentation significantly increased the content of all individual phenolic acids except for myricetin, which was slightly reduced. The addition of 0.3 g/L MP before alcohol fermentation significantly increased proanthocyanidin B2, caffeic acid, chlorogenic acid, catechin, trans-p-coumaric acid, and rutin content. It significantly reduced the content of proanthocyanidin B1, trans-ferulic acid, quercetin-3-D-β-glucoside, and myricetin. The addition of 0.1 g/L MP during storage significantly reduced the total amount of phenolic acids, and significantly reduced the content of proanthocyanidin B1, protocatechin, chlorogenic acid, catechin, trans-ferulic acid, quercetin-3-D-β-glucoside, myricetin, and quercetin, and had no significant effect on the contents of other phenolic acids. The addition of 0.3 g/L MP during storage significantly increased the total amount of phenolic acids and significantly increased the contents of gallic acid, proanthocyanidin B1, proanthocyanidin B2, quercetin-3-D-β-glucoside, and myricetin in the samples. However, it significantly reduced the content of chlorogenic acid, catechin, quercetin, and kaempferol. Our results were consistent with those of the predecessors, which found that the addition of MP after fermentation reduced the total phenolic acids content in wine (30). The results of this study show that the addition of MP before fermentation was more conducive to increasing the phenolic contents in wine. This might be because MP acted as a protective agent to reduce the degradation of phenolic acids during the fermentation process and played a protective role (14, 16).

**Table 3:** Effects of mannoprotein concentration on the individual phenolic acids profiles of wine at different stages of fermentation (mg/L).

**Anthocyanins**	**CK**	**Mannoprotein treatment (g/L)**			
		**Before fermentation**		**After fermentation**	
		**0.1**	**0.3**	**0.1**	**0.3**
Gallic acid	38.790 ± 0.27d	53.290 ± 0.08a	48.111 ± 0.56b	39.300 ± 0.14d	40.240 ± 0.26c
Proanthocyanidin B1	139.581 ± 0.78c	158.222 ± 0.31a	128.751 ± 0.91e	133.181 ± 0.67d	142.941 ± 0.98b
Protocatechin	11.470 ± 0.15b	14.050 ± 0.26a	11.460 ± 0.31b	10.990 ± 0.36c	11.600 ± 0.15b
Chlorogenic acid	34.130 ± 0.10c	42.280 ± 0.17a	37.671 ± 0.48b	32.280 ± 0.27d	32.570 ± 0.35d
Catechin	33.160 ± 0.77c	48.020 ± 0.03a	42.802 ± 0.70b	24.820 ± 0.04d	34.200 ± 0.05c
Proanthocyanidin B2	171.661 ± 0.82d	200.390 ± 0.21a	187.083 ± 0.69b	173.631 ± 0.51cd	175.782 ± 0.06c
Epicatechin	17.910 ± 0.29c	28.790 ± 0.61a	23.132 ± 0.52b	17.770 ± 0.64c	18.980 ± 0.20c
Caffeic acid	8.280 ± 0.09d	8.840 ± 0.07b	12.630 ± 0.16a	8.440 ± 0.04c	8.380 ± 0.00cd
Trans-*p*-coumaric acid	4.310 ± 0.46c	5.530 ± 0.80b	7.630 ± 0.34a	3.860 ± 0.07c	3.760 ± 0.08c
Rutin	12.761 ± 0.08b	18.052 ± 0.04a	18.180 ± 0.23a	13.090 ± 0.17b	13.930 ± 0.03b
Trans-ferulic acid	6.160 ± 0.07b	7.850 ± 0.05a	5.090 ± 0.01c	1.720 ± 0.20c	6.080 ± 0.07b
Quercetin-3-D-β-glucoside	25.800 ± 0.13c	32.930 ± 0.20a	22.091 ± 0.61d	15.830 ± 0.25e	29.110 ± 0.17b
Myricetin	7.480 ± 0.01b	6.970 ± 0.23c	5.110 ± 0.10d	4.470 ± 0.58e	8.460 ± 0.17a
Quercetin	9.250 ± 0.21b	10.490 ± 0.04a	9.170 ± 0.49b	5.890 ± 0.26c	7.250 ± 0.12c
Kaempferol	3.570 ± 0.03c	3.760 ± 0.01a	3.690 ± 0.00b	3.560 ± 0.01c	3.420 ± 0.02d
Total	524.31	639.46	562.59	488.83	536.7

*Different letters in the row indicate significant differences (Duncan test, p < 0.05) among treatments*.

*CK, control group with 0 g/L MP*.

### Antioxidant Activities of Wine Samples

**Figure 2:**
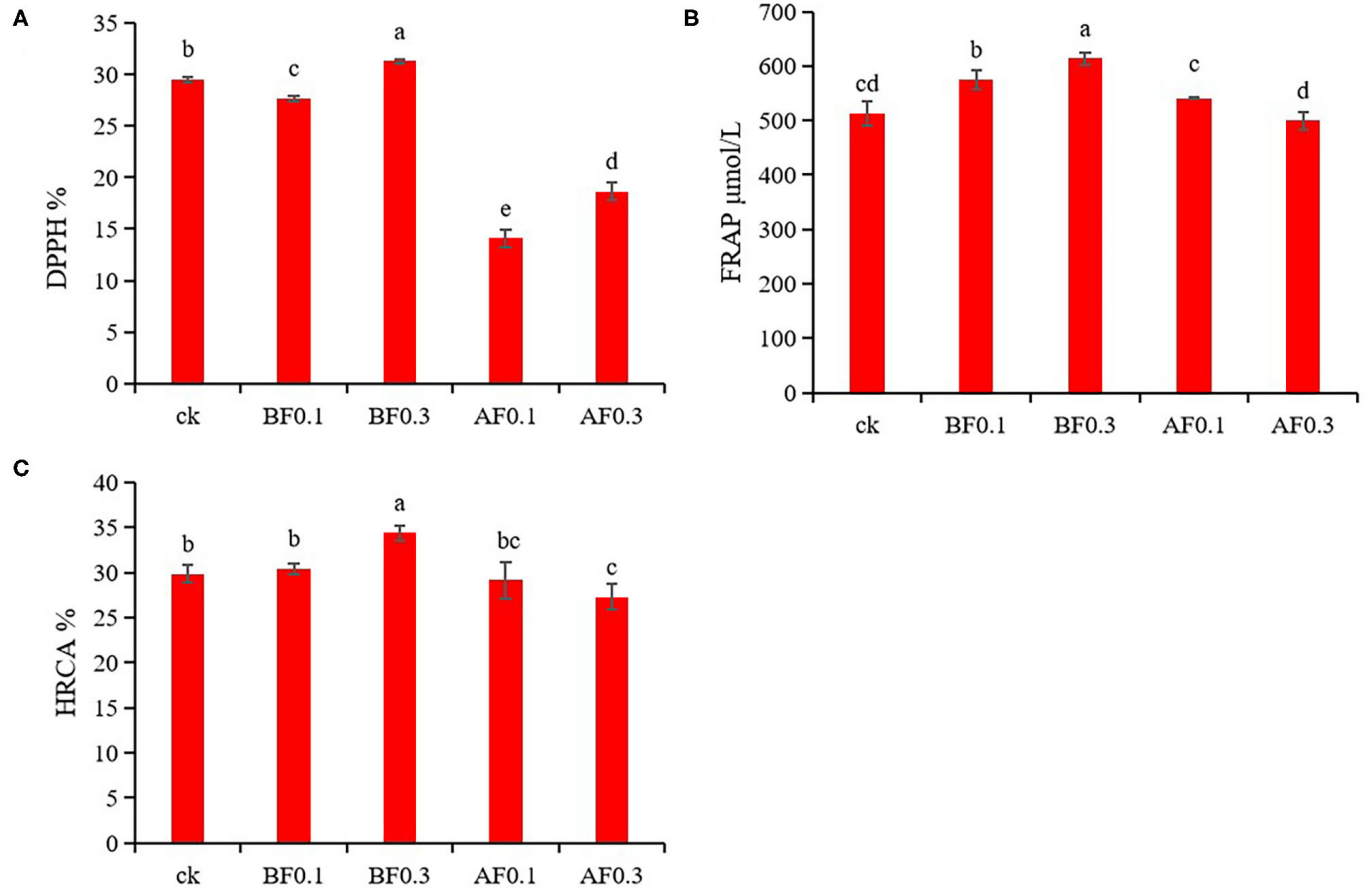
Effect of mannoprotein concentration on the antioxidant capacity of wine. **(A)**: 1,1-diphenyl-2-picrylhydrazyl (DPPH), **(B)**: ferric reducing antioxidant power (FRAP), **(C)**: hydroxyl radical scavenging ability (HRCA). Values presented are means ± SD (*n* = 3). Different letters indicate significant differences among treatments using Duncan test (*p* < 0.05). CK: control group with 0 g/L MP, BF0.1: 0.1 g/L mannoprotein addition before fermentation, BF0.3: 0.3 g/L mannoprotein addition before fermentation, AF0.1: 0.1 g/L mannoprotein addition after fermentation, BF0.3: 0.3 g/L mannoprotein addition after fermentation.

Wine is rich in phenolic acids and anthocyanins, and has strong antioxidant capacity, which is good for human health. This study analyzed the effects of the addition of MP on the phenolic acids and anthocyanins in wine. The results showed that the addition of MP could significantly affect the phenolic acids and anthocyanins in wine (Tables 2, 3). Hence, MPs likely have an impact on the antioxidant capacity of wine. The effects of MP addition on the antioxidant capacity of wine are shown in Figure 2. Compared with the control group, the addition of 0.1 g/L MP before fermentation significantly increased the FRAP value of wine samples to 574.31 μmol/L but reduced the DPPH value. The values of DPPH, FRAP, and HRCA were significantly increased in the group with 0.3 g/L MP before fermentation to 31.25%, 613.43 μmol/L, and 34.40%, respectively. These data indicate that the addition of 0.3 g/L MP before fermentation significantly improved the wine antioxidant capacity. Interestingly, the addition of MP during storage significantly reduced the DPPH, FRAP, HRCA values of wines, indicating that the addition of MP during storage reduced the antioxidant capacity of wines. The addition of MP before fermentation improved the antioxidant capacity of wine samples, which might be because the addition of MP increased the phenolic acids and anthocyanins in the wine (38).

### Multivariate Statistical Analysis

**Figure 3:**
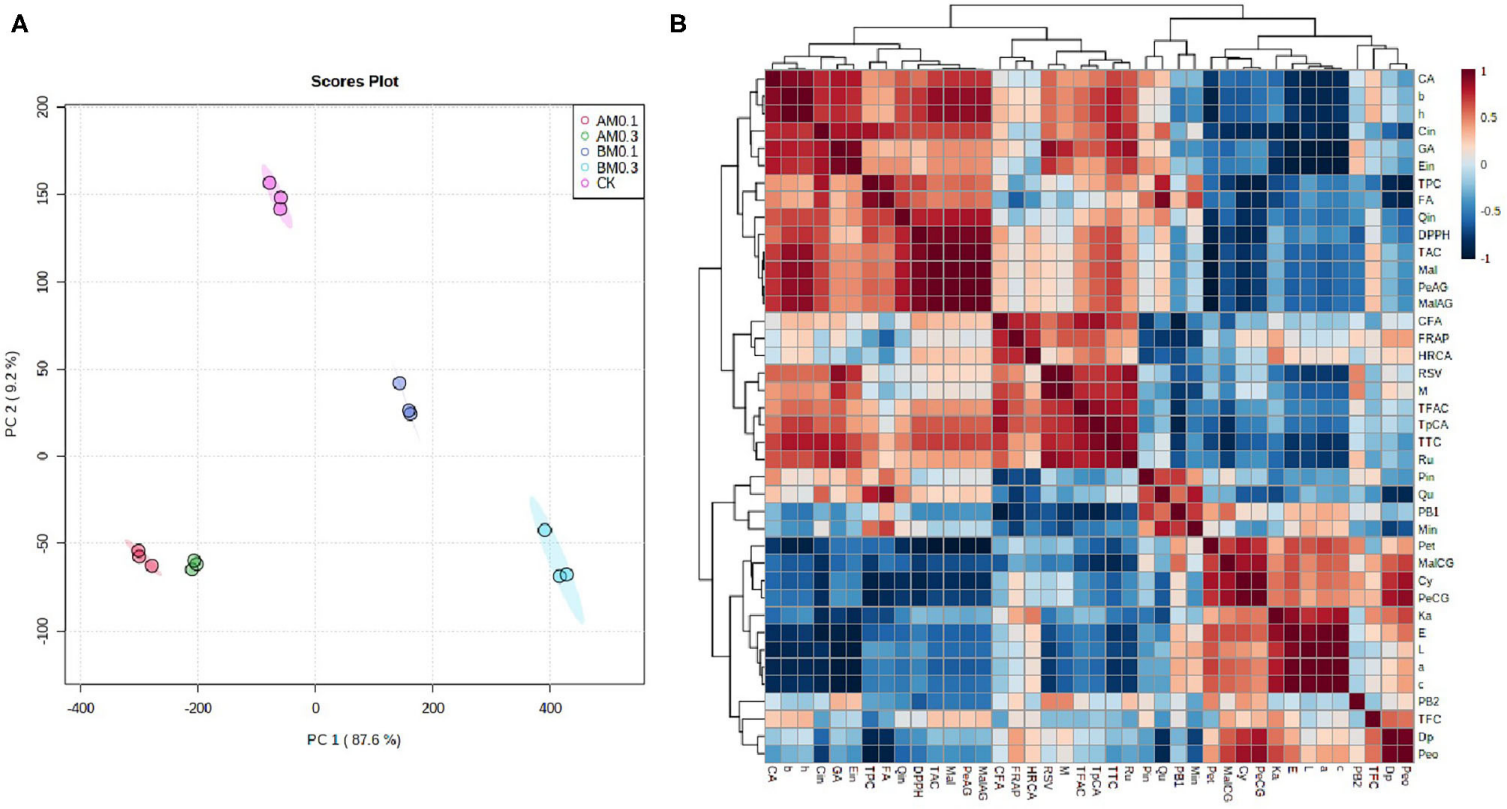
Heat maps of multivariate statistical analysis. **(A)** Principal components analysis (PCA). CK: control group with 0 g/L MP, BF0.1: 0.1 g/L mannoprotein addition before fermentation, BF0.3: 0.3 g/L mannoprotein addition before fermentation, AF0.1: 0.1 g/L mannoprotein addition after fermentation, BF0.3: 0.3 g/L mannoprotein addition after fermentation. **(B)** Correlation analysis between phenolics concentrations and color characteristics, antioxidant capacity. Data was normalized by a pooled sample from control groups.

Multivariate data analysis revealed the effects of MP addition on wine quality and functional components and analyzed the relationship between wine color stability, antioxidant capacity, anthocyanins, and phenolic acids. Figure 3A shows the principal components analysis (PCA) data. The first two principal components accounted for 96.8% (PC1 and PC2 were 87.6% and 9.2%, respectively). The MP treatment group and the control group were well distinguished, which indicated that the treatment effect was obvious. The treatment groups with MP added during storage were on the negative sides of PC1 and PC2. The distances were close, indicating that the effects of these two treatments on wine were consistent. The treatment groups with MP added before fermentation were located on the positive side of PC1. The 0.3 g/L MP treatment group was located on the negative side of PC2, and the 0.1 g/L MP treatment group was located on the positive side of PC2. These results were consistent with the results in Tables 2, 3.

Correlation analysis analyzed the relationship between anthocyanins, phenolic acids, color stability, and antioxidant capacity (Figure 3B). Wine color characteristics including ΔE, L^*^, a^*^, and C values were significantly positively correlated with the contents of petunidin-3-5-*O*-diglucoside, malvidin-3-*O*-coumayl-5-*O*-glucoside, cyanidin-3-5-*O*-glucoside, and peonidin-3-*O*-coumayl-5-*O*-glucoside. In addition, b^*^ and H values were significantly positively correlated with the contents of TAC, TPC, TTC, malvidin-3-5-*O*-diglucoside, petunidin-3-*O*-acetly-5-*O*-glucoside, malvidin-3-*O*-acetly-5-*O*-glucoside, catechin, gallic acid, epicatechin, and rutin. The addition of MP could significantly change the contents of these anthocyanins and phenolic acids in wine samples (Tables 2, 3). Previous studies found that the color stability was directly related to the content of anthocyanins and phenolic acids (20). These results showed that the addition of MP—especially addition before fermentation—affected the color stability of wine by affecting the contents of anthocyanins and phenolic acids in wine. In addition, the results demonstrated that the antioxidant capacity of wine including DPPH, FRAP, and HRCA values had a significantly positive correlation with TPC, TAC, TTC, TFAC, rutin, trans-p-coumaric acid, quercetin, epicatechin, gallic acid, catechin, and gallic acid contents. There was a significant positive correlation between polyphenols and antioxidant activity (39, 40). The results of this study were consistent with previous studies, and these data indicated that the addition of MP affected the antioxidant capacity of wine by affecting the phenolic content in wine samples (38).

## Conclusions

This study investigated the effects of mannoprotein (MP) addition at crushing and storing on anthocyanins, phenolic acids, color characteristics, and antioxidant activities during viticulture. The results showed that the addition of MP before alcohol fermentation could significantly increase the content of anthocyanins and phenolic acids. The addition of MP during storage had a negative impact on the anthocyanin contents in wine, but the addition of 0.3 g/L MP during storage could increase the phenolic contents. The results showed that the addition of MP before fermentation could better improve the color stability and antioxidant capacity of wine, which in turn was beneficial to the long-term storage of wine and improved the nutritional value of wine.

## Data Availability Statement

The original contributions presented in the study are included in the article/Supplementary Material, further inquiries can be directed to the corresponding authors.

## Author Contributions

YLJ, ZWZ, and YLF designed the research and provided materials. SSJ, XFN, and XFY, conducted the experiments. YLJ and SSJ analyzed the data. YLJ and XFY wrote the manuscript. KKZ promoted the manuscript. All the authors have read and approved the final manuscript.

## Conflict of Interest

The authors declare that the research was conducted in the absence of any commercial or financial relationships that could be construed as a potential conflict of interest.
