# Supplementary figures and images for: Anthocyanin and Phenolic Acids Contents Influence the Color Stability and Antioxidant Capacity of Wine Treated With Mannoprotein
Source: Front Nutr. 2021 Jun 18;8:691784. doi: 10.3389/fnut.2021.691784 (PMC8249586; doi:10.3389/fnut.2021.691784)

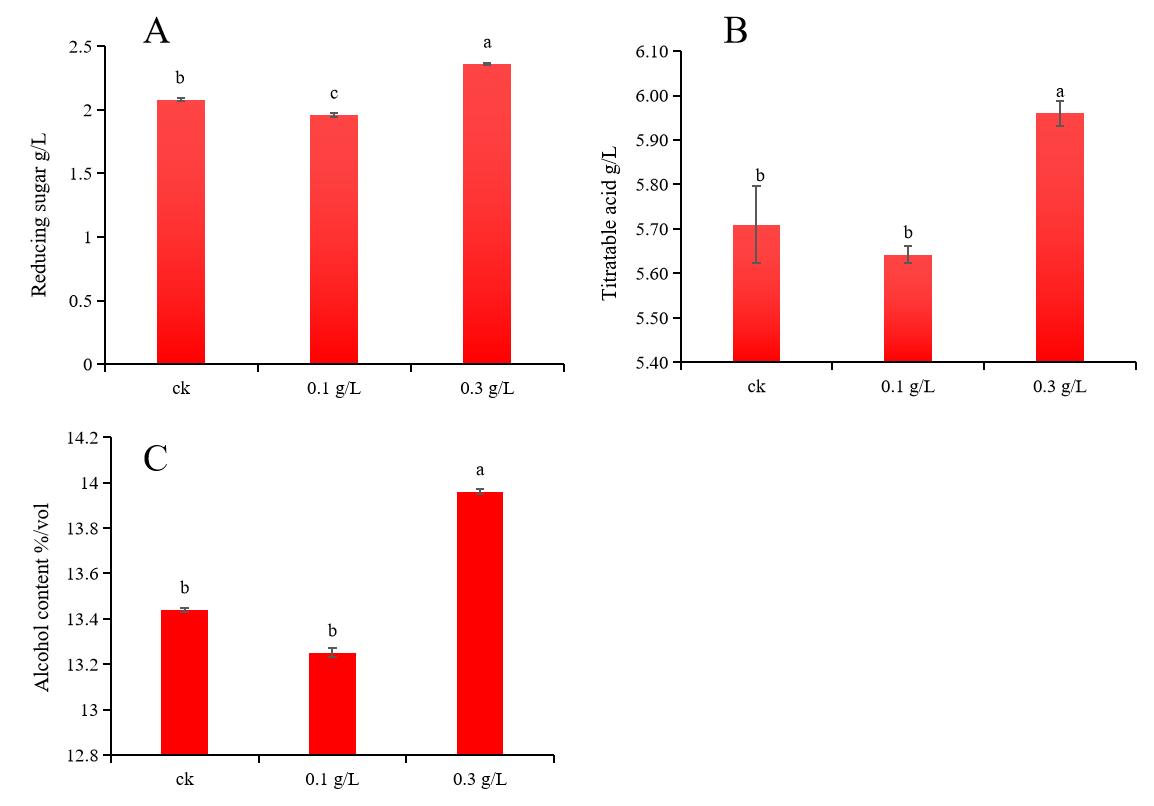

Supplement: Supplementary file 1 [file Image_1.jpg]

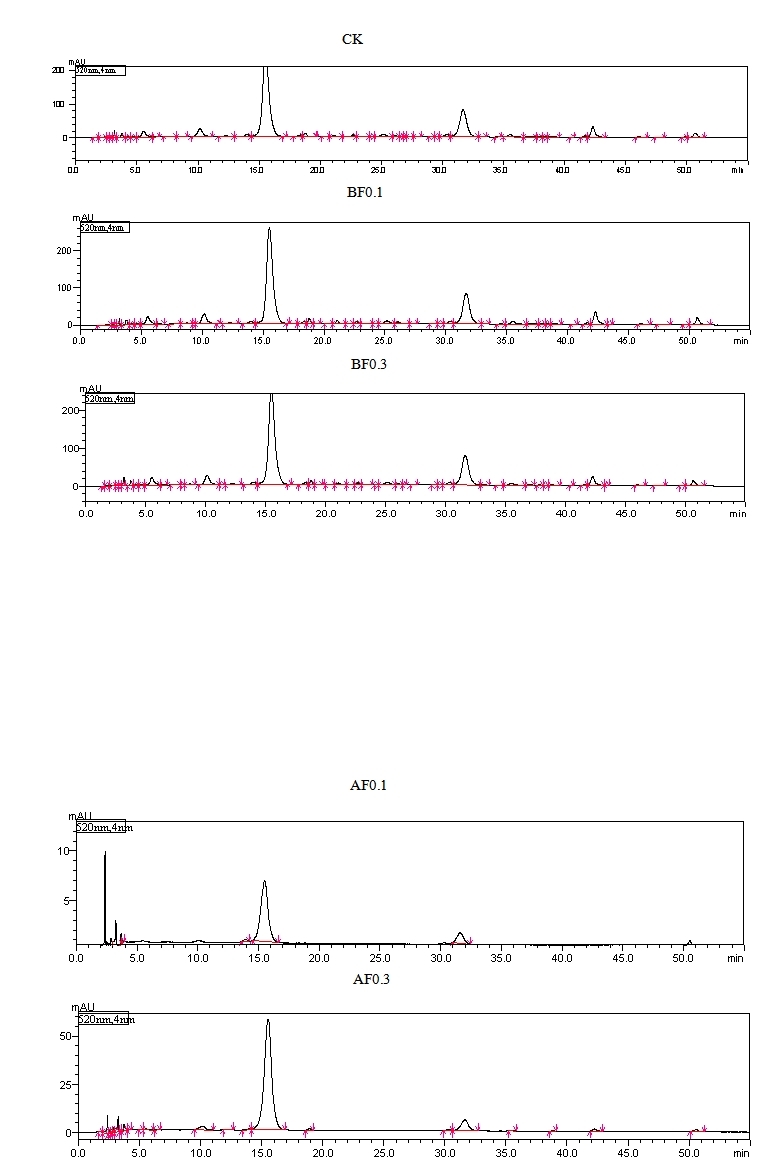

Supplement: Supplementary file 2 [file Image_2.jpg]

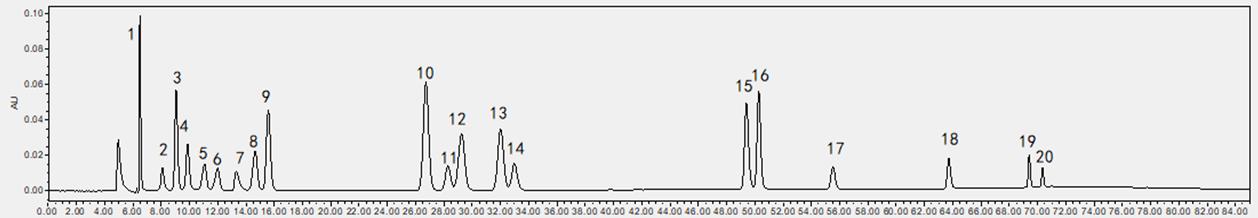

Supplement: Supplementary file 3 [file Image_3.jpg]
